# Current status of umbilical cord blood storage and provision to private biobanks by institutions handling childbirth in Japan

**DOI:** 10.1186/s12910-022-00830-8

**Published:** 2022-09-12

**Authors:** Maho Murata, Kenichiro Kawabe, Taichi Hatta, Shoichi Maeda, Misao Fujita

**Affiliations:** 1 Course for Health Care Management and Public Health, Graduate School of Health Management, Keio University, 4411 Endo, Fujisawa-shi, Kanagawa 252-0882 Japan; 2 Graduate School of Public Health, Shizuoka Graduate University of Public Health, Aoi-ku, Shizuoka, 420-0881 Japan; 3 Uehiro Research Division for iPS Cell Ethics, Center for iPS Cell Research and Application, Kyoto University, 53 Kawahara-cho, Shogoin, Sakyo-ku, Kyoto, 606-8507 Japan; 4 Institute for the Advanced Study of Human Biology (WPI-ASHBi), Kyoto University, Yoshidakonoe-cho, Sakyo-ku, Kyoto, 606-8501 Japan

**Keywords:** Umbilical cord blood, Private biobank, Public biobank, Questionnaire, Japan

## Abstract

**Background:**

The Act Regarding the Promotion of the Appropriate Supply of Hematopoietic Stem Cells for Transplant regulates only how public banks store and provide umbilical cord blood (UCB) for research or transplantation. Japan had no laws to regulate how the private banks manage the procedures, harvesting, preparation, and storage of such blood. As a result, the status of UCB distribution remains unknown. We conducted a survey to investigate the current status of UCB storage and provision to private biobanks by Japanese institutions that handle childbirth.

**Methods:**

Questionnaire forms were mailed to 3,277 facilities handling childbirth that were registered in the Japan Council for Quality Health Care website.

**Results:**

Of the 1,192 institutions handling childbirth that participated in the survey (response rate: 36.7%), 34.4% responded that they currently provide UCB to private biobanks, while 16.1% of facilities did so in the past. Moreover, some institutions currently provide or formerly provided UCB to medical treatment facilities (2.6%), research institutions (5.9%), companies (2.2%), or overseas treatment facilities, research institutions, or companies (0.3%). A certain number of institutions handling childbirth did not even provide explanations or obtain consent when the UCB was harvested from private bank users.

**Conclusions:**

This is the first study to determine the status of UCB provision to private banks by Japanese institutions handling childbirth. Future studies will need to examine in detail how institutions handling childbirth provide explanations to private bank users and UCB providers as well as how these institutions obtain consent.

**Supplementary Information:**

The online version contains supplementary material available at 10.1186/s12910-022-00830-8.

## Background

In 1998, the first umbilical cord blood (UCB) transplant was performed between blood relatives of a patient with Fanconi anemia [1]. Since then, more than 35,000 UCB transplants have been carried out worldwide [2]. Furthermore, UCB is a rich source of mesenchymal stem cells that may be used in regenerative medicine. Clinical trials are being conducted to determine the effectiveness of hematopoietic stem cells and mesenchymal stem cells in the management of progressive cerebral palsy and autism, and other nervous system and autoimmune diseases such as type 1 diabetes mellitus [3].

The importance of UCB has also been increasing in line with technological developments in regenerative medicine. Although UCB was once regarded as medical waste and disposed in the past, its utility in both clinical and research contexts is now increasing because it is not only used in hematopoietic stem cell transplants but also regarded as a raw material for basic research using stem cells such as human induced pluripotent stem cells (hiPSCs) [4, 5].

Because UCB can be harvested only once in a person’s life, that is, at the time of birth, the amount of harvestable UCB is limited. Thus, public and private banks that store UCB have been established worldwide. Currently, the UCB of approximately 800,000 individuals is stored in public banks and that of more than five million individuals in private banks [6].

Public banks collect and store donated UCB for use in hematopoietic stem cell transplants between unrelated individuals in the future. They operate on public funding, and while donors do not bear any financial costs, they also do not have any control over how and for whom their UCB is used. UCB is usually donated in hospitals that are officially affiliated with public banks. Private banks, run by for-profit companies, store UCB so that the donors and/or their families can use it in the future if it is needed for the treatment of a disease. In general, donors and/or their families pay for the expense of storage and use the UCB for themselves.

In Japan, the first public bank for UCB storage was launched in 1999. Supply of UCB to such banks is authorized by the Ministry of Health, Labour, and Welfare (MHLW), and the procedures for collecting, harvesting, preparing, and storing of such blood are regulated by the “Act Regarding the Promotion of the Appropriate Supply of Hematopoietic Stem Cells for Transplant (APHSCT).” For therapeutic purposes, the public bank can supply blood only to the medical facility that performs UCB transplants only for 27 conditions (including Hodgkin’s disease, acute leukemia, and so forth). Transplants in such facilities accounted for 1,496 (cumulative total of 19,737) in 2020 [7], and as of September 2021, the number of patients for whom UCB was stored was 9,436 [7]. Based on the APHSCT, public banks were used to supply UCB only to improve the safety and effectiveness of hematopoietic stem cell transplants and the prevention and treatment of diseases. After the discovery of hiPSCs in 2007 [8], stem cell-based interventions that utilize hiPSCs have drawn widespread attention. To differentiate various cells from iPSCs that can be used for as many patients as possible, hiPSC lines derived from HLA homozygote donors, whose blood types are quite rare, are needed. The APHSCT was amended in 2014, which made it possible to find HLA homozygote donors and collect and use their UCB stored in public banks for the production of iPSCs for research and for therapy in the future [9, 10].

However, there are no clear set of regulations followed by the Japanese private banks regarding the procedures for collecting, harvesting, preparing, and storing UCB. This is because the APHSCT is applicable only to public banks. The Act on the Safety of Regenerative Medicine (ASRM) promulgated in 2014 regulates UCB transplants mediated by private banks regardless of the purpose is research or therapy. Under the law, researchers and physicians should report the details of their interventions. However, the ASRM does not have information on how private banks carry out the procedures for collecting, harvesting, preparing, and storing such blood. Moreover, currently, there are no data in Japan about the collection source for UCB and the collection procedure used by private banks for storing UCB specimens. Currently, unproven interventions utilizing UCB that claim to treat various diseases have been reported and deemed problematic [11, 12]. However, the mode and method for collecting, storing, and distributing such blood remains unclear. Therefore, we conducted a survey to delineate the actual status of UCB storage, focusing on Japanese medical institutions that handle childbirth.

## Methods

### Samples and attributes

The survey target was 3,277 facilities registered in the Japan Council for Quality Health Care (JCQHC) website for childbirth (hospital or clinic = 2,835, maternity center = 442) (as of January 18, 2017). According to information registered in the website, 99.9% of childbirth facilities were members of the JCQHC at that time in Japan [13]. On March 17, 2017, a request for participation letter, a questionnaire form, and a stamped self-addressed envelope were posted to 3,277 institutions and a request was made to the relevant managers to respond to the questionnaire anonymously and return it by post. Furthermore, a reminder card was sent to all institutions on April 20, 2017. The last response was received on June 12, at which point data collection was concluded. Return of the questionnaire form was considered to constitute consent to participate in the study.

### Questionnaire content

We prepared a questionnaire concerning UCB storage and distribution. While this paper specifically reports the following questions, these are only a subset of a more comprehensive questionnaire that includes other items (see Additional files 1 and 2).

Explanation of UCB and banking: An explanation of UCB and banking was provided, followed by question items below:

Question 1: “At present, does your institution handle childbirth?” Respondents who answered “Yes” went to Question 2 and those who answered “No” went to Question 5.

Question 2: “With regard to UCB, do you carry out the following measures?” Respondents provided information on several measures as follows: supply to public banks; supply to private banks; supply directly to treatment facilities; supply directly to research institutions; supply directly to companies; supply to overseas treatment facilities, research institutions, or companies; store in-house for treatment purposes; store in-house for research purposes; and other measures. For each measure, respondents were asked to choose one of the three options such as “Currently do,” “Done in the past, but not now,” or “Never.”

Question 3: “How does your institution provide explanations when obtaining informed consent for harvesting UCB?” Respondents were asked to choose one of three options, “Explanation methods are standardized,” “Explanation methods differ according to purpose,” or “No explanation is provided.”

Question 4: “How does your institution obtain informed consent when harvesting UCB?” Respondents were asked to choose one of three options, “Methods of obtaining consent are standardized,” “Methods of obtaining consent adopt to purpose,” or “Consent is not obtained.”

Question 5: Respondents were asked about the type of institution (hospital designated for clinical training, hospital not designated for clinical training, medical clinic, or maternity center), sex, and age.

### Data analysis

Descriptive analysis of the data was done for all the responses. The statistical analysis software package SPSS, version.22, was used in the analysis.

## Results

Of the 3,277 questionnaires distributed, 25 were not delivered because the institution no longer existed or the address was revised or unknown. The total number of responses was 1,192 (response ratio: 36.7%). By institution type, responses came from 360 hospitals designated for clinical training (30.2%), 126 hospitals not designated for clinical training (10.6%), 511 medical clinics (42.9%), and 182 maternity centers (15.3%). Thirteen institutions that responded did not answer about the type of institution (1.1%). The respondents’ sex and age are shown in Table 1. Overall, 745 respondents were male and 421 were female. As for age, 269 were in their 40's , 465 in their 50's, and 289 in their 60's.

**Table 1:** Respondents’ sex and age (N = 1,192)

*Sex*	n(%)
Male	745(62.5)
Female	421(35.3)
Do not want to answer	11(0.9)
No response	15(1.3)
*Age group, years*	
20–29	7(0.6)
30–39	71(6.0)
40–49	269(22.6)
50–59	465(39.0)
60–69	289(24.2)
70–79	50(4.2)
80-	11(0.9)
Do not want to answer	18(1.5)
No response	12(1.0)

Question 1: In total, 1,084 respondents answered that their institutions handled childbirth (91.0%), 98 respondents answered that their institutions did not handle childbirth (8.2%), and 10 respondents provided no information (0.8%).

Question 2: Of the 1,084 respondents whose institutions currently handle childbirth, 153 respondents answered “Currently do” or “Done in the past, but not now” regarding UCB supply to public banks (14.1%) (Table 2). The number of those who answered “Currently do” or “Done in the past, but not now” to other measures were as follows: supply to private banks (548/50.4%); supply directly to treatment facilities (28/2.6%); supply directly to research institutions (64/5.9%); supply directly to companies (23/2.1%); supply to overseas treatment facilities, research institutions, or companies (3/0.3%); store in-house for treatment purposes (25/2.3%); store in-house for research purposes (34/3.1%); and other measures (e.g., sending for assessment of pH, SpO_2_ or SpCO_2_, giving it back to mother, etc.) (41/3.4%).

**Table 2:** Management of umbilical cord blood at institutions that currently handle childbirth n(%)

	Currently do	Done in the past, but not now	Never	No answer
Provide to a public blood bank	66(6.1)	87(8.0)	879(81.1)	52(4.8)
Provide to a private blood bank	373(34.4)	175(16.1)	503(46.4)	33(3.0)
Provide to a medical treatment facility	13(1.2)	15(1.4)	1001(92.3)	55(5.1)
Provide to a research institution	25(2.3)	39(3.6)	967(89.2)	53(4.9)
Provide to a company	17(1.6)	6(0.6)	1007(92.9)	54(5.0)
Provide to a foreign medical treatment facility, research institution, or company	2(0.2)	1(0.1)	1026(94.6)	55(5.1)
Store at own facility for medical treatment purposes	10(0.9)	15(1.4)	1005(92.7)	54(5.0)
Store at own facility for research purposes	19(1.8)	15(1.4)	994(91.7)	56(5.2)
Other measures	32(2.7)	9(0.8)	664(55.7)	379(31.8)

Question 3: Of the 548 respondents whose institutions had current or past experiences to provide UCB to private banks, 253 answered that their explanation methods were standardized (46.2%), 101 answered that their explanation methods differed according to purpose (18.4%), 143 respondents answered that they did not provide explanation (26.1%), and 51 respondents did not answer the question (9.3%).

Question 4: Of the 548 respondents whose institutions had current or past experiences to provide UCB to private banks, 251 answered that they had a uniform method for obtaining consent (45.8%), 106 respondents answered that they had methods of obtaining consent to adopt purpose (19.3%), 101 answered that they did not obtain consent (18.4%), and 90 did not answer the question (16.4%).

## Discussion

In the present study, we derived two particularly noteworthy results. First, nearly half of the institutions that responded to the study were either currently providing UCB to private banks during the study period or had done so in the past. Second, some institutions were found to provide UCB not only to private banks but also to companies, research institutions, and medical treatment facilities.

### Provision of UCB to private banks by institutions handling childbirth

During the present study, the APHSCT, along with related ministerial ordinances and guidelines, stipulated how public banks preserve and manage UCB. However, during the study period, these laws and regulations did not require the institutions that handled childbirth to keep records, except when providing UCB to public banks. Consequently, no one knew how many institutions handling childbirth supplied UCB to private banks or the status of UCB distribution. The present study determined that 34.4% of institutions handling childbirth currently provide UCB to private banks, while 16.1% of institutions did so in the past. Our study reported for the first time that these percentages far outstrip those for UCB supply to public banks (6.1% and 8.0%, respectively). These low percentages may be related to the low number of institutions handling childbirth in Japan partnered with public banks (96 institutions as of January 18, 2021) [14–19].

However, from the standpoint of appropriate collection, safe preservation, and effective usage of UCB, public and private banks should be regulated according to more uniform standards. More than one-fourth of institutions that provide or have provided UCB to private banks did not provide explanations about UCB collection to UCB donors, while nearly 20% of institutions did not obtain consent. Donors of UCB choose to have their UCB preserved and are also users of UCB who entrust their UCB to private banks, a state of affairs that may lead to the opinion that it is not that important for institutions handling childbirth to provide explanations or obtain consent. However, an MHLW survey reported that private banks do not provide sufficient explanations to users in advance [20]. This state of affairs may be related to the absence of regulations in private banks in Japan.

Even before we demonstrated problems with private banks in Japan in the present study with empirical data, these problems were already known anecdotally, which led many academic associations to issue warnings. In 2002, the Japan Society for Hematopoietic Cell Transplantation issued a statement declaring that private banks were almost completely ineffective, except in cases such as patients with refractory blood diseases within one’s own family and that regulations were necessary to ensure proper technical guidelines and safety [21]. In addition, the Japan Association of Obstetricians and Gynecologists declared in 2002 that sufficient understanding was necessary regarding the status and background of private storage of UCB and that careful steps were required to ensure that private banks do not simply use UCB for profit [22].

However, as we analyzed the results of the present study, a relevant concern came to pass. In 2017, physicians who administered UCB to patients without notifying government authorities were found guilty of violating the Act on the Safety of Regenerative Medicine, with the vendor who sold the UCB charged as an accomplice [23, 24]. The UCB sold by the vendor leaked from a private bank that had gone bankrupt in 2009. However, the charge in this case was providing regenerative medicine to patients without reporting it to the MHLW; there was no law targeting the sale of the leaked UCB itself, which was, therefore, beyond the scope of legal penalty [25].

Spurred by the case described above, the MHLW conducted a survey of private UCB banks in Japan [20]. Of the seven vendors whose activities could be confirmed at the time of the survey, six responded; one of these vendors only distributed UCB without preserving it. The UCB held by the remaining five vendors constituted a supply for a total of 45,800 people; roughly 2,100 people’s worth of UCB had not been disposed after the vendors’ contracts with the donors had ended. One vendor provided UCB to a third party (roughly 160 times). The three vendors involved in the above case later went out of business [26].

Taking the case seriously, the MHLW revised the APHSCT to generally prohibit the collection, preparation, storage, testing, and delivery of UCB for transplantation as a business by entities other than public banks. The revision also stipulated that UCB for transplantation may not be delivered by anyone for commercial purposes. However, these prohibitions do not apply when a public bank delivers UCB, when UCB is used in the treatment of a blood relative to the donor, or when approval is granted by the MHLW. Violations of these prohibitions are subject to criminal penalties. Consequently, the two private banks that obtained approval from the MHLW were permitted to continue their activities.

However, regardless of legal permission, there is still the question of whether private UCB banks, which handle UCB for profit, are ethically permissible. For example, the 2004 European Commission’s Group on Ethics in Science and New Technologies stated that while they did not completely disavow for-profit biobank activities, these activities engender ethical criticism. The group also stated that the human body in principle is not an object of commercial value and recommended that private biobank activities operate under strict conditions such as appropriate management by regulatory authorities [27]. Meanwhile, a non-Japanese study has reported that the possibility of UCB being used 20 years later by the person who requested its preservation or by their family is an incredibly low 0.04–0.0005% [28]. The extent to which this information is explained to potential private bank users is unknown. In fact, the previously cited survey by the MHLW indicated that the role of public UCB banks and the actual utility of the UCB stored in the private banks were not sufficiently explained to users [20]. Future research must thoroughly examine the status of UCB private banks following revision of the law and compare the results of this examination to the findings of the present study.

### Provision of UCB from institutions handling childbirth to institutions other than private banks and UCB storage

A small number of institutions handling childbirth surveyed in the present study responded that they currently provide or used to provide UCB to medical treatment facilities (2.6%), research institutions (5.9%), companies (2.2%), or foreign medical treatment facilities, research institutions, or companies (0.3%). Some institutions handling childbirth also either currently store or used to store UCB themselves for treatment or research (2.3% and 3.2%, respectively). This aspect of the status of UCB distribution has never been demonstrated in a previous study.

Since the revision of the APHSCT, the delivery of UCB for transplantation has been strictly prohibited except in the cases of provision to a public bank, provision to a private bank approved by the MHLW, and use for treatment by a blood relative. Thus, it is currently considered illegal for institutions handling childbirth to deliver UCB to other facilities domestically or internationally or to store UCB themselves for treatment purposes. However, the revised law still does not apply to the handling of UCB for research purposes, that is, basic studies and the development of treatments. In addition, while there are laws and local ordinances that call for the incineration or burial of UCB according to specific methods, these regulations generally do not cover the delivery of UCB for research purposes.

At a glance, there would seem to be no problem with an institution that handles childbirth providing UCB to a third party or storing UCB itself for research purposes. However, the results of the present study, which found that a certain number of institutions handling childbirth do not provide explanations or obtain consent when UCB is harvested from private bank users, and the results of the above-cited MHLW survey, which found that private banks also fail to provide users with sufficient explanations, cast doubt amidst the absence of relevant laws and regulations as to how much has been suitably explained to UCB donors when they consent to be third-party UCB donors.

We did not determine what sort of explanations institutions handing childbirth give when they deliver UCB to other institutions or store it themselves for research purposes, nor did we determine methods for obtaining consent, as we felt these fell outside the aim of the present study. Future studies must answer these questions and evaluate if there truly is no problem with the current state of affairs in Japan in the absence of rules regarding the harvest or delivery of UCB for research purposes by institutions handling childbirth.

### Limitations and prospects

The present study had several limitations. First, the response rate was only 36.7%, which is not at all high. However, the percentages of institutions handling childbirth by type that responded to our survey are roughly consistent with those of Japanese medical treatment facilities overall [29], implying that our results are representative to some extent. Of course, we cannot rule out the effect of non-responder bias. However, the present study can be considered sufficiently significant because this is the first study to determine the status of UCB delivery by Japanese institutions handling childbirth to private banks, other companies, research institutions, and medical treatment facilities. The 3,277 facilities included in this study represent 99.9% of childbirth facilities in Japan. The total number of facilities in Japan is approximately 3,280. Of which 1,084 facilities responded that they handled childbirth. A simple calculation from the actual number of births in 2016 (976,978 births), a year before this study was conducted [30], allowed us to estimate that the facilities included in our study handled a total of 322,879 births. The number of UCBs managed by these facilities can be considered significant. In addition, by determining the status of UCB delivery prior to revision of the APHSCT, we have made it possible to determine the effects of APHSCT via comparisons with post-revision survey results.

## Conclusions

Future studies will need to examine in detail how institutions handling childbirth provide explanations to private bank users and UCB providers as well as how these institutions obtain consent. The status of harvesting and delivery of UCB, a limited resource, must be further elucidated so that it can be utilized more effectively and safely.

## Supplementary Information

**Additional file 1**: Information on umbilical cord blood and umbilical cord blood banks.

**Additional file 2**: Survey on the handling of umbilical cord blood.

## Data Availability

No additional data available.

## Abbreviations

UCB: Umbilical cord blood
hiPSCs: Human induced pluripotent stem cells
MHLW: Ministry of Health, Labour, and Welfare
APHSCT: Act Regarding the Promotion of the Appropriate Supply of Hematopoietic Stem Cells for Transplant
ASRM: Act on the Safety of Regenerative Medicine
JCQHC: Japan Council for Quality Health Care
