# Supplementary material for: Current status of umbilical cord blood storage and provision to private biobanks by institutions handling childbirth in Japan
Source: BMC Med Ethics. 2022 Sep 12;23:92. doi: 10.1186/s12910-022-00830-8 (PMC9465943; doi:10.1186/s12910-022-00830-8)
Supplement: Supplementary file 1 — Additional file 1: Information on umbilical cord blood and umbilical cord blood banks. [file 12910_2022_830_MOESM1_ESM.docx]

**Information on Umbilical Cord Blood and Umbilical Cord Blood Banks**

○Introduction

This study concerns umbilical cord blood banks. The following document explains what umbilical cord blood is, why it is sometimes stored, and how umbilical cord blood banks work.

○What is umbilical cord blood?

Umbilical cord blood is the blood that flows through the umbilical cord that connects a baby and its mother. This blood has the same DNA type as the baby and does not mix with the mother’s blood under normal circumstances. It is only possible to harvest umbilical cord blood at birth, when the umbilical cord between the newborn baby and its mother is cut. In other words, childbirth is the only time in life when umbilical cord blood can be collected. If not collected and stored at this time, it is generally disposed of.

○Hematopoietic stem cells in umbilical cord blood

Hematopoietic stem cells perform the function of making blood within the bone marrow that exists in bones. Umbilical cord blood is rich in these cells. If hematopoietic stem cells stop functioning, blood diseases such as leukemia and aplastic anemia can develop.

A hematopoietic stem cell transplant is a way of treating such blood diseases by transplanting healthy hematopoietic stem cells harvested from a donor to reestablish the ability to produce healthy blood.

Furthermore, hematopoietic stem cells have the potential to be of use in the still-developing field of regenerative medicine, for example, material for producing induced pluripotent stem cells (iPSCs).

○Hematopoietic stem cell transplants/HLA type

Hematopoietic stem cells possess a human leukocyte antigen (HLA) type. If HLA types do not match, the hematopoietic stem cells will not provide treatment well even if they are transplanted. Therefore, it is necessary to match the HLA types of the donor and patient.

However, the probability of finding someone with the same HLA type ranges from one in several hundreds to several tens of thousands. In the case of siblings born to the same parents in a single family, there will be four types of HLA, meaning that the probability of having matching HLA types will be one in four. Therefore, most patients who require a transplant do not have a person with the same HLA type in their family.

○Storage of umbilical cord blood

As noted above, umbilical cord blood is not only useful for hematopoietic stem cell transplants that are currently available, it can also be used in research into treatments such as regenerative medicine that may be used in the future. Furthermore, there is the possibility that it will be a part of future treatments that are not yet proven to be effective. However, umbilical cord blood can only be harvested in a particular situation and thus must be stored correctly.

When the new parents desire to preserve their child’s umbilical cord blood, it is common practice for the childbirth facility to harvest the blood in an appropriate way and then pass it to an umbilical cord bank for storage. In Japan, two types of umbilical cord blood banks exist: public banks and private banks.

Public banks supply umbilical cord blood as public works. At present, the Japanese Red Cross Society supports the operation of hematopoietic stem cell supply services, with six such banks operating in Japan. Private banks are umbilical cord banks that are operated by private companies.

○Storing umbilical cord blood in public banks

To store umbilical cord blood in a public bank, (1) the mother must give birth in a medical institution that is affiliated with a public bank and (2) the umbilical cord blood that the medical institution collects and passes to the bank must meet certain standards, such as the required cell count. Stored umbilical cord blood is supplied to applicable patients for use in hematopoietic stem cell transplants, without the donor and his/her family receiving any preferential usage rights. In general, umbilical cord blood is stored for ten years.

In recent times, expired umbilical cord blood or that which does not meet the required standards has been used in research in fields such as regenerative medicine.

○Storing umbilical cord blood in private banks

The mother gives birth at a medical institution that is affiliated with a private bank, the institution collects the umbilical cord blood from the newborn baby and passes it on to the bank, and the bank stores it. The blood will be stored so that the individual whose blood is donated or their close family can use it for hematopoietic stem cell transplants or for possible regenerative treatments in the future. What happens to the umbilical cord blood in the case when the storage period expires or it does not meet the required standards differs from bank to bank.

Table: Characteristics of public and private banks

|  | Who Can Use Donated Cells | Use of Cells | Cost to Donors | Harvesting Institution |
| --- | --- | --- | --- | --- |
| Public Bank | General patients deemed to require treatment (no priority given to donor or donor’s family) | Transplant (donors cannot choose who will use their cells) and Research | No | 88 institutions in Japan |
| Private Bank | Donor and their families, etc., who may fall ill in the future | Transplant (the donor or their close family) and Research | Yes^[[1]](#footnote-1)^ | Details unclear  (Estimated to be more than several hundred) |

○Storage without using a bank

At present, it is not against the law for medical institutions to collect umbilical cord blood and store it in their own facilities or to supply it to other institutions (e.g. hospital, research institution, or companies) without working through an intermediary bank.

As discussed earlier, umbilical cord blood is usually discarded after birth, but it can be used, if necessary, in the following ways.

Umbilical cord blood donated to a public bank can be used for the treatment of others if it meets the required standards and for research purposes if it does not. Blood supplied to a private bank can be used for the treatment of the child or his/her family in case of an illness in the future. Additionally, it can be used for the treatment of others (excluding umbilical cord blood transplants) if directly supplied to a treatment facility, for research purposes if supplied to a research institution, and for the production and sale of medical products if supplied to a company.

1. For example, JPY210,000 (initial fee + 10 years storage) [↑](#footnote-ref-1)
