# Supplementary material for: Current status of umbilical cord blood storage and provision to private biobanks by institutions handling childbirth in Japan
Source: BMC Med Ethics. 2022 Sep 12;23:92. doi: 10.1186/s12910-022-00830-8 (PMC9465943; doi:10.1186/s12910-022-00830-8)
Supplement: Supplementary file 2 — Additional file 2: Survey on the handling of umbilical cord blood. [file 12910_2022_830_MOESM2_ESM.docx]

Survey on the Handling of Umbilical Cord Blood

　Question 1. Storage, Donation, and Use of Umbilical Cord Blood

Suppose that you or your partner is going to give birth. Please answer the following questions.

1. Of the following possible methods, which one would you prefer for the handling of your child’s umbilical cord blood? Please tick the box next to the one answer that most closely matches your preference.

□ 1. I would like to donate it to a public blood bank for umbilical cord blood transplants for others and/or research purposes.

□ 2. I would like to save it in a private blood bank for use if the child or his/her family becomes ill in the future.

□ 3. I would like to supply it directly to a treatment facility for the treatment (excluding umbilical cord blood transplants) of others in procedures that are not covered by insurance.

□ 4. I would like to supply it directly to a research institution for research purposes.

□ 5. I would like to supply it to a company for the purpose of manufacturing pharmaceuticals.

□ 6. None of the above (1-5).

(2) “If your child’s umbilical cord blood is not being used by the child or his/her family,, would you like to donate it for the purpose of research or the treatment of others, as in the cases below? For each question (a-f) below, tick the one box that most closely matches your feelings.

|  | 1.No | 2.Not Really | 3.Somewhat | 4.Yes |
| --- | --- | --- | --- | --- |
| a. Research for increasing the safety and effectiveness of umbilical blood transplants in the future (e.g., effective ways of separating hematopoietic stem cells) |  | | | |
| b. Research into areas other than existing umbilical cord blood transplants, for the development of new preventative techniques or treatments (e.g., iPS cell research) |  | | | |
| c. Treatments that are covered under current insurance  (e.g., umbilical cord blood transplants for treating leukemia, etc.) |  | | | |
| d. Treatments that are not currently covered by insurance  (e.g., treatment of cerebral palsy, etc.) |  | | | |
| e. Treatments that are not for the purpose of curing people with illnesses (e.g., beauty or anti-aging treatments, etc.) |  | | | |
| f. For the manufacture and sale of pharmaceuticals  (e.g., treatment of acute graft versus host disease, etc.) |  | | | |

(3) For each question (a-o) below, please tick the one box that most closely matches your feelings.

|  | 1.No | 2.Not Really | 3.  Somewhat | 4.Yes |
| --- | --- | --- | --- | --- |
| a. I would like the child’s umbilical cord blood to be of value to society. |  | | | |
| b. I would like the child’s umbilical cord blood to be of use to people with illnesses. |  | | | |
| c. I feel hesitant to decide how my child’s umbilical cord blood will be handled. |  | | | |
| d. There is hope for new types of treatment such as regenerative medicine. |  | | | |
| e. I think that parts of the human body should not be used for profit-making purposes. |  | | | |
| f. We have a duty to help patients that are in life-threatening situations. |  | | | |
| g. I am resistant to the idea of my own umbilical cord blood being used by others. |  | | | |
| h. I am resistant to the idea of using the umbilical cord blood of others should I need it. |  | | | |
| i. Institutions approved by the state can be trusted. |  | | | |
| j. I am wary of preserving or donating/supplying umbilical cord blood. |  | | | |
| k. I would like to know about the disadvantages associated with the supply and storage of umbilical cord blood. |  | | | |
| l. I am worried about stored or supplied umbilical cord blood being used for unintended purposes. |  | | | |
| m. I have concerns about the safety of personal information related to stored or supplied umbilical cord blood. |  | | | |
| n. Paperwork for storing or supplying umbilical cord blood is bothersome. |  | | | |
| o. There should not be a financial cost associated with the storage or supply of umbilical cord blood. |  | | | |

　Question 2. Regarding Ownership of Umbilical Cord Blood

(1) Who owns umbilical cord blood? Please circle all numbers that match what you think.

1. Child　　2. Mother　　3. Father　　4. No one　　5. Other（　　　　　　　　　）

(2) Suppose that you or your partner donate your child’s umbilical cord blood to a public bank for the purpose of umbilical cord blood transplant treatment or research. Suppose that ten years later, advances in medical technology make it possible for the umbilical cord blood that you donated to be used for research in regenerative medicine that were not thought possible at the time of your donation. In such a case, do you think that the public bank should be required to contact you, your partner, or your child and reobtain consent? Please tick the one box that matches your feelings.

□1. Yes　　　　 　 Why? Please circle all numbers that match what you think.

1. Because I/they have the right to know

2. Because I feel uncomfortable with it being used without my/their knowledge

3. Because my/their feelings may have changed in the ten years

4. Because I want to know about the way personal information is handled

5. Because umbilical cord blood belongs to the child

6. Because I would like to have a good discussion with our child about it

7. Other (　　　　　　　　　　　　　　　　　　　　　　　　　)

From whom should the new consent be sought? Please circle all numbers that match what you think.

1. Child　　2. Mother　　3. Father　　4. Other (　　　　　　　　　　)

□2. No 　　 　 Why? Please circle all numbers that match what you think.

　　　　　　　　　　1. Because it would hinder the development of regenerative medicine

2. Because the umbilical cord blood belongs to the public bank

3. Because the paperwork would be bothersome

4. Other (　　　　　　　)

　Question 3. Handling of Umbilical Cord Blood at your Medical Institution

Please answer the following for the case of your medical institution.

(1) At present, does your institution handle childbirth? Please tick the one box that matches your situation.

□ 1. Yes　　　　　　　□ 2. No (→Please go to Question 4）

(2) With regard to umbilical cord blood, do you carry out the following measures? For each question (a-j) below, please tick the one box that most closely matches your situation.

|  | 1. Never | 2. Done in the past but not now | 3. Currently do |
| --- | --- | --- | --- |
| a. Supply to public banks | □ | □ | □ |
| b. Supply to private banks | □ | □ | □ |
| c. Supply directly to treatment facilities | □ | □ | □ |
| d. Supply directly to research institutions | □ | □ | □ |
| e. Supply directly to companies | □ | □ | □ |
| f. Supply to overseas treatment facilities, research institutions, or companies | □ | □ | □ |
| g. Store in-house for treatment purposes | □ | □ | □ |
| h. Store in-house for research purposes | □ | □ | □ |
| i. Other measures (　　　　　　　　　　　　　　) | □ | □ | □ |
| j. Umbilical cord blood is not collected | □ | □ | □ |

Please circle all numbers that are applicable as reasons why umbilical cord blood is not being

collected.

1. We do not have the necessary facilities in place. 2. The financial burden is too large.

3. The burden on staff is too much. 4. There are no public banks nearby.

5. There are no private banks nearby. 6. Other (　　　　　　　　　　　）

(3) How does your institution provide explanations when obtaining informed consent for harvesting umbilical cord blood? Please tick the one box that matches your situation.

□ 1. Explanation methods are uniform. □ 2. Explanation methods differ according to purpose.

□ 3. No explanation provided.

Please circle all numbers that match the explanation methods that are provided.

1. Posters are displayed. 2. Pamphlets are provided. 3. Verbal explanations are given.

4. A record of the explanation is taken. 5. Written materials are given in person.

(4) How does your institution obtain informed consent when harvesting umbilical cord blood? Please tick the one box that matches your situation.

□ 1. Methods of obtaining consent are uniform.　□ 2. Methods of obtaining consent differ according to purpose. □ 3. Consent is not obtained.

　　 Please circle the one box that matches your situation.

□1. Verbal consent with no record made □2. Verbal consent with record made

□3. Written consent obtained

Please circle all numbers that match who you obtain consent from.

1. Mother　2. Father　3. Differs according to the purpose 4. Other（　　　　　　　　　）

　Question 4. Regarding regulations for handling of umbilical cord blood other than in public blood banks.


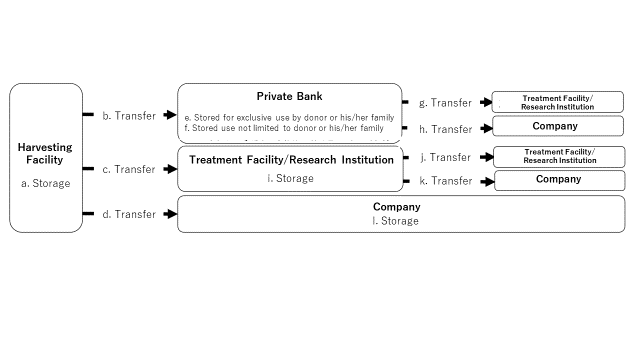
(1) With regard to handling of umbilical cord blood by entities other than public banks and whether various activities should be engaged in and/or regulated, please answer the following, thinking about your own feelings. The following cases are based on the premise that the action is in line with the wishes of the donor. For each question (a-l) below please tick the one box which most closely matches your own feelings.

|  | 1. The state should regulate | 2. Academic associations, etc., should regulate | 3. Relevant institution should self-regulate | 4. Should be free from regulation |
| --- | --- | --- | --- | --- |
| a. Storage by the harvesting facility | □ | □ | □ | □ |
| b. Transfer from the harvesting facility to a private bank | □ | □ | □ | □ |
| c. Transfer from the harvesting facility to a treatment facility or research institute | □ | □ | □ | □ |
| d. Transfer from the harvesting facility to a company | □ | □ | □ | □ |
| e. Storage by a private bank (for use only by the donor or his/her family) | □ | □ | □ | □ |
| f. Storage by a private bank (for use by the donor, his/her family or others) | □ | □ | □ | □ |
| g. Transfer from a private bank to a treatment facility or research institution | □ | □ | □ | □ |
| h. Transfer from a private bank to a company | □ | □ | □ | □ |
| i. Storage by a treatment facility or research institution | □ | □ | □ | □ |
| j. Transfer from a treatment facility or research institution to another treatment facility or research institution | □ | □ | □ | □ |
| k. Transfer from a treatment facility or research institution to a company | □ | □ | □ | □ |
| l. Storage by a company | □ | □ | □ | □ |

(2) For each question (a-j) below, please tick the one box that most closely matches your own feelings.

|  | 1. No | 2. Not Really | 3. Somewhat | 4. Yes |
| --- | --- | --- | --- | --- |
| a. State or administrative institutions should take a broad approach to managing the circulation of umbilical cord blood. |  | | | |
| b. There are ethical issues concerning the circulation of umbilical cord blood. |  | | | |
| c. Responsibilities with regard to the handling of umbilical cord blood should be made clear. |  | | | |
| d. Umbilical cord blood should be provided to those who need it. |  | | | |
| e. Those who wish to donate umbilical cord should be able to do so freely. |  | | | |
| f. Treatment that uses umbilical cord blood should be developed further |  | | | |
| g. Research that uses umbilical cord blood should be developed further |  | | | |
| h. The personal information of the donor should be not be given with the umbilical cord blood, ensuring anonymity. |  | | | |
| i. Where umbilical cord blood is stored in a private blood bank, priority usage rights for the child and his/her family should be guaranteed by law. |  | | | |

　Question 5. Please answer these questions about yourself

| a. Location of Institution | Prefecture: |
| --- | --- |
| b. Type of Institution | □1. Hospital designated for clinical training  □2. Hospital other than above.  □3. Medical Clinic  □4. Maternity center |
| c. Gender | □1. Male　　□2. Female　　□3. Prefer not to say |
| d. Age | □1. 20s　 □2. 30s　 □3. 40s　　 □4. 50s  □5. 60s　 □6. 70s　 □7. 80 and over　□8. Prefer not to say |
| e. Have you ever had a blood transfusion? | □1. Yes　　□2. No　　□3. Prefer not to say |
| f. Have you ever donated blood? | □1. Yes　　□2. No　　□3. Prefer not to say |
| g. Are you registered as a bone marrow donor? | □1. Yes　　□2. No　　□3. Prefer not to say |
| h. Are you an organ donor? | □1. Yes　　□2. No　　□3. Prefer not to say |
| i. Have you ever stored umbilical cord blood in a private bank? | □1. Yes　　□2. No　　□3. Prefer not to say |
| j. Have you donated umbilical cord blood to a public bank? | □1. Yes　　□2. No　　□3. Prefer not to say |
| k. Have you or your partner ever given birth? | □1. Yes　　□2. No　　□3. Prefer not to say |

　Free Comment Space

Thank you. Your cooperation is greatly appreciated.
